# Depression, anxiety, stress, social interaction and health-related quality of life in men and women with unexplained chest pain

**DOI:** 10.1186/1471-2458-8-165

**Published:** 2008-05-19

**Authors:** Annika Janson Fagring, Karin I Kjellgren, Annika Rosengren, Lauren Lissner, Karin Manhem, Catharina Welin

**Affiliations:** 1 The Sahlgrenska Academy at Göteborg University, Institute of Health and Care Sciences, Göteborg, Sweden; 2 Department of Medicine, Sahlgrenska University Hospital/Östra, Göteborg, Sweden; 3 Department of Community Medicine and Public Health/Epidemiology, the Sahlgrenska Academy, Göteborg University, Sweden; 4 Department of Medicine, Sahlgrenska University Hospital/Mölndal, Göteborg, Sweden

## Abstract

**Background:**

Unexplained chest pain (UCP) is a common reason for emergency hospital admission and generates considerable health-care costs for society. Even though prior research indicates that psychological problems and impaired quality of life are common among UCP patients, there is lack of knowledge comparing UCP patients with a reference group from the general population. The aim of this study was to analyse differences between men and women with UCP and a reference group in terms of psychosocial factors as depression, anxiety, stress, social interaction and health-related quality of life (HRQOL).

**Methods:**

A self-administered questionnaire about psychosocial factors was completed by 127 men and 104 women with acute UCP admitted consecutively to the Emergency Department (ED) or as in-patients on a medical ward. A reference group from the general population, 490 men and 579 women, participants in the INTERGENE study and free of clinical heart disease, were selected.

**Results:**

The UCP patients were more likely to be immigrants, have a sedentary lifestyle, report stress at work and have symptoms of depression and trait-anxiety compared with the reference group. After adjustment for differences in age, smoking, hypertension and diabetes, these factors were still significantly more common among patients with UCP. In a stepwise multivariate model with mutual adjustment for psychosocial factors, being an immigrant was associated with a more than twofold risk in both sexes. Stress at work was associated with an almost fourfold increase in risk among men, whereas there was no independent impact for women. In contrast, depression only emerged as an independent risk factor in women. Trait-anxiety and a low level of social interaction were not independently associated with risk in either men or women. Patients with UCP were two to five times more likely to have low scores for HRQOL.

**Conclusion:**

Both men and women with UCP had higher depression scores than referents, but an independent association was only found in women. Among men, perceived stress at work emerged as the only psychosocial variable significantly associated with UCP.

## Background

Unexplained chest pain (UCP) is a common reason for emergency hospital admission and generates considerable health-care costs for society [1,2]. Previous studies have often defined UCP as non-cardiac, i.e. chest pain that had not been diagnosed as acute myocardial infarction (AMI) or ischemic heart disease (IHD) by a physician [3]. Galmiche et al [4], has developed three diagnostic criteria for functional chest pain of presumed esophageal origin i.e. "1) Midline chest pain or discomfort that is not of burning quality. 2) Absence of evidence that gastroesophageal reflux is the cause of the symptom. 3) Absence of histopathology-based esophageal motility disorders" (p. 1461). Psychological factors e.g. anxiety and somatisation disorders will also have an impact on the functional chest pain. However, Fox [5] stresses that the terminology "unexplained chest pain", i.e. chest pain, which after investigation has proven to be unrelated to the heart, might be confusing but is still preferable to "non-cardiac" or "atypical chest pain".

Keogh et al. [6] reported that women with high anxiety sensitivity report more chronic pain than women with low anxiety sensitivity. No similar relationship was found for men. Gender differences are also seen in the diagnosis of chest pain, with male patients more likely than women to be diagnosed with cardiac chest pain instead of non-cardiac chest pain [6], in accordance with their higher overall risk of IHD.

Studies of patients with a non-cardiac/non-coronary diagnosis of chest pain often include patients with other defined causes of chest pain, e.g. gastro-esophageal and musculoskeletal disorders [7]. However, a considerable number of the patients with a non-cardiac/coronary diagnosis do not have a clearly defined explanation for their chest pain.

Psychological problems are often reported in patients with unexplained chest pain. In a study of predictors of health-care seeking among non-cardiac chest pain patients, Eslick et al. [3] found that the main reason for seeking care was anxiety, with 78% of patients with non-cardiac chest pain having sought health care in the last 12 months. Previous qualitative studies in UCP patients have found that the physical, psychological and social consequences of chest pain disturbed and negatively affected the patient's daily life [8,9].

Even though prior research indicates that psychological problems and impaired quality of life are common among UCP patients, there is lack of knowledge comparing UCP patients with a reference group from the general population. Because psychological symptoms are prevalent also among the general population, comparing UCP patients and a reference group might improve knowledge of the importance of these factors in the increasingly large group of patients suffering from UCP.

The aim of the present study was to analyse differences between men and women with UCP and a reference group in terms of psychosocial factors as depression, anxiety, stress and health-related quality of life (HRQOL).

## Methods

The study was carried out at Sahlgrenska University Hospital in Göteborg, Sweden. The study was approved by the Ethics Committee at Göteborg University, Sweden (study code 169-02), and was carried out in accordance with the Declaration of Helsinki.

### Study sample, setting and procedure

#### The patients

Data were collected during office hours, Monday through Friday, from December 2002 to September 2003. Patients between 25–69 years of age, who were 1) evaluated for acute chest pain and judged by a physician to have no organic cause of their chest pain and 2) free from any history of heart disease were considered for inclusion in the study. In all, 648 patients with UCP were eligible at ED during the study period. However, because patients could only be investigated during office hours and not during holidays or weekends only 337 patients were considered for inclusion during the study period. Patients who were too ill to participate (n = 4) or had language difficulties (n = 37) were excluded. Eighteen patients declined to participate and 47 could not be investigated for administrative reasons (e.g. an overcrowded ED). In all, 231 patients (127 men and 104 women) took part in the study (78%). Of these 157 were investigated in the ED and the remainder (n = 74) after being admitted to a medical ward for further investigation. The results of the cardiac investigation were not known to the patient at the time of the interview. All the eligible patients were assessed and treated according to the routines of the ED. They were asked about participation by the investigators, thereafter written and verbal information about all the steps in the study was provided. After written informed consent was obtained, the patients filled in a questionnaire providing background characteristics and data on psychosocial factors, before being discharged. The ED patients filled in the questionnaire shortly after arrival in hospital, the in-patients within 24 hours after arrival.

#### The reference group

Residents of Göteborg aged 25–69 years and free of clinical heart disease were recruited from the INTERGENE study. INTERGENE is a population-based study assessing the interplay between genetic susceptibility, environmental factors, lifestyle and psychosocial background as risk factors for chronic diseases and cardiovascular disease. The reference group consisted of all the participants in the INTERGENE study. Of the recruited sample (n = 2,422), 1,477 (61%) came to the screening during the study period. Of these, 28 (17 men and 11 women) with previous MI/angina were excluded. Episodes (current or former) of chest pain were not investigated. The HRQOL and psychosocial questionnaires were introduced in a secondary survey of participants in the primary survey. For this reason, 380 people (189 men and 191 women) did not complete the questionnaires because they did not participate in the secondary survey. In all, 1,069 individuals (490 men and 579 women) participated (74% of those screened without previous MI/angina). The reference participants were assessed according to the protocol for the INTERGENE study [10]. The psychological assessment was conducted after the basic examination.

#### Measurements

All the scales measuring psychosocial factors and HRQOL [11-15], are presented in Table 1. *Perceived stress at work *was assessed with one item (response alternatives; (0) never perceived stress, (1) some period of stress, (2) some period of stress during the last five years, (3) several periods of stress during the last five years, (4) permanent stress during the last year, (5) permanent stress during the last five years).

**Table 1:** Measurement scales used in the study and their reliability.

**Scale**	**Assessment**	**Items**	**Scale score**	**Reliability**	**References**
Interview Schedule	Social integration	6	0–30	Test-retest reliability 0.75	Henderson et al. 1980
for Social Interaction	Social attachment	8	0–16	Test-retest reliability 0.76	Undén et al. 1989
Zung Self-Rating	Symptoms of depression	20	20–80	Chronbach's alpha 0.92	Zung 1965
Depression Scale					
Trait-Anxiety	Trait anxiety	20	20–80	Chronbach's alpha 0.83 to 0.92	Spielberg 1968
Inventory					
SF 36	Health-related quality of life	36	0–100	Chronbach's alpha 0.79 to 0.93	Sullivan, Karlsson 1994

The patients' *perception of their marriage or cohabitation *was assessed with two items, i.e. "How do you perceive your marriage or cohabitation?" (response alternatives; (1) very happy, (2) fairly happy, (3) difficult to say, (4) rather unhappy, (5) very unhappy; summery score: 2–10), and "How often do you have difficulty getting along with your wife or husband or cohabitant?" (response alternatives; (1) never, (2) seldom, (3) sometimes, (4) very often, (5) almost all the time; summery score 2–10) [16].

Demographics and background characteristics from the questionnaire were recorded from both the UCP patients and the reference group members regarding *age*; *marital status *(single, married/cohabitating, divorced, widow/er); *education *(compulsory/secondary school/university); *work status *(employed full time/part time, early retirement/disability pension, full time/part time, retired, unemployed, others); *Immigrant status *was assessed by a question of country of birth. Not born in Sweden was in present study defined as immigrant; *physical activity in leisure time *(response alternatives; (1) sedentary in leisure time, (2) moderate exercise in leisure time (walking, riding bicycle, light gardening for a minimum of 4 hours), (3) regular exercise and training (strenuous activity for a minimum of 3 hours/week), (4) intense training or competitive sport); *physician confirmed diabetes *(yes/no); *physician confirmed hypertension *(yes/no); *current smoker *(yes/no); *ex-smoker *(yes/no); *alcohol consumption *(response alternatives including; frequency and amount each week/month/day). *Weight and height *were self-reported by UCP patients but measured in reference group at the screening examination.

### Statistical analyses

The analyses were carried out using the Statistical Analysis System (SAS) 8.2 (SAS Institute Inc., Cary, NC). Differences between the UCP patients and the referents in terms of demographic and psychosocial factors were tested by the chi-square test for discrete variables and by Student's t-test for continuous variables. Odds ratios were calculated for selected background characteristics and psychosocial variables simultaneously controlling for age, smoking, hypertension and diabetes. Multivariate logistic stepwise regression models were used. In step 1, all variables were included if p < 0.10 in the univariate analysis. In step 2, variables were included if p < 0.10 in step 1. A two-sided p ≤ 0.05 was considered significant.

## Results

Demographics and background characteristics in the UCP patients and the reference group are shown in Table 2. The mean age of the UCP men was significantly lower (45.7 years) compared with the male referents (48.7 years). Twenty five per cent of the UCP women had attended university, compared with 45% of the referents. Differences in education for men were less pronounced. The UCP patients, particularly the UCP women, had more hypertension compared with the female referents (p < 0.0001). BMI was significantly higher in both UCP men and women than in the reference group. Current smoking was more common among UCP women than among their referents, whereas there were no differences in reported smoking among the men. In contrast, reported alcohol consumption/week was significantly lower in the UCP patients, among both men and women.

**Table 2:** Demographic and background characteristics in UCP patients (127 men, 104 women) and referents (490 men, 579 women).

	UCP		Referents		
			
	%	(n)	%	(n)	p-value
**MEN**					
Age, years, mean (SD)	45.7	(11.1)	48.7	(12.3)	0.01
Single	24	(31)	28	(135)	0.45
University education	28	(35)	38	(184)	0.04
Employed	71	(90)	72	(348)	0.87
Current smoker	16	(20)	17	(84)	0.70
Diabetes^a^	4	(5)	5	(24)	0.68
Hypertension^a^	20	(25)	14	(67)	0.07
Body mass index, mean (SD)	27.4	(3.5)	26.5	(3.6)	0.01
Alcohol consumption g/week, mean (SD)	32.7	(25.6)	48.8	(34.5)	<.0001
					
**WOMEN**					
Age, years, mean (SD)	47.7	(11.9)	47.1	(12.2)	0.68
Single	30	(31)	35	(199)	0.33
University education	25	(26)	45	(259)	0.0001
Employed	60	(62)	69	(398)	0.05
Current smoker	34	(35)	22	(125)	0.01
Diabetes^a^	6	(6)	1	(6)	0.001
Hypertension^a^	30	(30)	12	(72)	<.0001
Body mass index, mean (SD)	26.7	(5.9)	25.1	(4.4)	0.01
Alcohol consumption g/week, mean (SD)	18.0	(25.7)	28.2	(20.0)	0.0002

^a ^Physician confirmed

The percentage of immigrants was significantly higher among the UCP patients and these patients were also significantly more sedentary during their leisure time, compared with the reference group (Table 3). Twice as many men and three times as many women were immigrants among the UCP patients in comparison with the reference group. After adjustment for age, smoking, hypertension and diabetes, a significantly increased risk persisted (OR 2.6 (1.7–4.2), 3.7 (2.2–6.3)), in men and women respectively. A sedentary lifestyle was also significantly associated with UCP after adjustment for age, smoking, hypertension and diabetes (OR 2.9 (1.7–4.9) and 2.0 (1.1–3.7) for men and women respectively).

**Table 3:** Comparison between 231 UCP patients (127 men, 104 women) and 1069 referents (490 men, 579 women).

	UCP		Referents				Adjusted^†^	
				
	%	(n)	%	(n)	OR	(95% CI)	OR	(95% CI)
**MEN**								
Immigrant^a^	33	(42)	16	(78)	2.6	(1.7–4.0)	2.6	(1.7–4.2)
Sedentary in leisure time	25	(31)	11	(54)	2.6	(1.6–4.3)	2.9	(1.7–4.9)
Perceived stress at work^b^	35	(36)	12	(55)	3.9	(2.4–6.4)	3.5	(2.1–6.0)
Mental strain in the marriage/cohabitation, score ≥ 7^c^	7	(9)	4	(19)	1.9	(0.8–4.3)	2.0	(0.9–4.5)
Depression, score ≥ 39^d^	35	(43)	18	(87)	2.4	(1.6–3.8)	2.3	(1.4–3.6)
Trait-anxiety, score ≥ 43^d^	36	(44)	19	(89)	2.4	(1.6–3.7)	2.2	(1.4–3.5)
Low social integration, score ≤ 9^e^	21	(26)	20	(97)	1.1	(0.7–1.8)	1.2	(0.7–2.0)
Low social attachment, score ≤ 11^f^	16	(20)	23	(109)	0.6	(0.4–1.1)	0.7	(0.4–1.3)
								
**WOMEN**								
Immigrant^a^	31	(32)	11	(63)	3.7	(2.3–6.1)	3.7	(2.2–6.3)
Sedentary in leisure time	20	(20)	9	(52)	2.5	(1.4–4.4)	2.0	(1.1–3.7)
Perceived stress at work^b^	32	(25)	18	(96)	2.1	(1.3–3.6)	2.0	(1.1–3.4)
Mental strain in the marriage/cohabitation, score ≥ 7^c^	10	(9)	5	(26)	2.2	(1.0–4.9)	2.3	(1.0–5.3)
Depression, score ≥ 39^d^	50	(50)	28	(159)	2.6	(1.7–4.1)	2.5	(1.6–3.9)
Trait-anxiety, score ≥ 43^d^	47	(47)	30	(169)	2.1	(1.4–3.3)	1.8	(1.1–2.8)
Low social integration, score ≤ 9^e^	32	(31)	20	(112)	2.0	(1.2–3.1)	1.8	(1.1–2.9)
Low social attachment, score ≤ 11^f^	11	(11)	16	(90)	0.6	(0.3–1.3)	0.5	(0.3–1.1)

^† ^Adjusted for age, smoking, hypertension, and diabetes; ^a ^Not born in Sweden; ^b ^Constant during the last year or the last five years; ^c ^Two items, total score 2–10; ^d ^Highest quintile in the total referent group, scale score 20–80; ^e ^Lowest quintile in the total referent group, scale score 0–30; ^f ^Lowest quintile in the total referent group, scale score 0–16;

UCP patients reported significantly more stress at work, symptoms of depression and trait-anxiety than the reference group (Table 3). After adjusting for age, smoking, hypertension and diabetes, these factors were still significantly more common among patients with UCP for both men and women; perceived stress at work OR 3.5 (2.1–6.0) and 2.0 (1.1–3.4), for men and women respectively; symptoms of depression OR 2.3 (1.4–3.6) and 2.5 (1.6–3.9); and trait-anxiety OR 2.2 (1.4–3.5) and 1.8 (1.1–2.8). Mental strain in marriage/cohabitation and low social integration only emerged as significant risk factors among women, after adjustment for age, smoking, hypertension and diabetes OR 2.3 (1.0–5.3) and OR 1.8 (1.1–2.9) respectively.

Table 4 presents a multivariate model in which significant background variables and psychosocial factors were taken into account. Being an immigrant emerged as an independent risk factor in both men (OR 2.05 (1.15–3.66)) and women (OR 2.76 (1.56–4.86)), while alcohol consumption also retained its independent significance (OR 0.98 (0.97–0.99), OR 0.97 (0.95–0.99) for men and women respectively. After adjustment, stress at work was associated with an almost fourfold increase in risk among men (OR 3.94 (2.26–6.85)), whereas there was no independent impact for women. In contrast, university education was an independent protective factor among women, with an OR of 0.47 (0.28–0.79). A sedentary lifestyle was associated with a twofold risk (OR 2.00 (1.06–3.79)), but only in men, whereas depression only emerged as an independent risk factor in women (OR 2.09 (1.30–3.38)). Trait-anxiety and low social interaction were not independently associated with risk in either men or women.

**Table 4:** Comparison between UCP patients and referents in multivariate analyses. In step 1, all variables are included if p < 0.10 in univariate analysis. In step 2, variables are included if p < 0.10 in step 1.

**MEN**	**Step 1**			**Step 2**		
				
	OR	(95% CI)	p-value	OR	(95% CI)	p-value
Age, per year	0.95	(0.93–0.98)	<0.0001	0.96	(0.94–0.98)	<0.0001
University education, yes/no	0.69	(0.40–1.18)	0.18			
Immigrant^a^, yes/no	1.76	(0.95–3.26)	0.07	2.05	(1.15–3.66)	0.02
Hypertension^b^, yes/no	1.32	(0.62–2.80)	0.46			
Body mass index	1.04	(0.97–1.11)	0.32			
Sedentary at leisure time, yes/no	1.81	(0.93–3.51)	0.08	2.00	(1.06–3.79)	0.03
Alcohol consumption, g/week	0.98	(0.97–0.99)	<0.0001	0.98	(0.97–0.99)	<0.0001
Perceived stress at work^c^	2.69	(1.47–4.95)	0.001	3.94	(2.26–6.85)	<0.0001
Depression, score ≥ 39^d^	1.52	(0.71–3.28)	0.28			
Trait-anxiety, score ≥ 43^e^	1.17	(0.53–2.56)	0.70			
Low social attachment, score ≤ 11^f^	0.50	(0.25–1.00)	0.05	0.54	(0.28–1.03)	0.06

**WOMEN**	**Step 1**			**Step 2**		
				
	OR	(95% CI)	p-value	OR	(95% CI)	p-value

University education, yes/no	0.45	(0.24–0.84)	0.01	0.47	(0.28–0.79)	0.004
Employed, yes/no	2.06	(0.97–4.38)	0.06	1.36	(0.81–2.30)	0.24
Immigrant^a^, yes/no	2.56	(1.25–5.24)	0.01	2.76	(1.56–4.86)	0.0005
Current smoker, yes/no	1.54	(0.82–2.90)	0.18			
Diabetes^b^, yes/no	2.43	(0.46–12.79)	0.29			
Hypertension^b^, yes/no	2.12	(1.01–4.44)	0.05	2.30	(1.30–4.05)	0.004
Body mass index	1.02	(0.96–1.09)	0.47			
Sedentary at leisure time, yes/no	1.71	(0.73–4.02)	0.22			
Alcohol consumption, g/week	0.98	(0.96–1.00)	0.02	0.97	(0.95–0.99)	0.002
Perceived stress at work^c^	1.62	(0.83–3.15)	0.16			
Mental strain in the marriage/cohabitation, score ≥ 7^g^	0.94	(0.31–2.88)	0.92			
Depression, score ≥ 39^d^	2.07	(0.97–4.44)	0.06	2.09	(1.30–3.38)	0.002
Trait-anxiety, score ≥ 43^e^	0.93	(0.43–2.02)	0.86			
Low social integration, score ≤ 9^h^	0.81	(0.40–1.62)	0.55			

^a ^Not born in Sweden; ^b ^Physician confirmed; ^c ^Constant during the last year or the last five years; ^d ^Zung depression scale, score 20–80; ^e ^Trait-anxiety inventory, score 20–80; ^f ^ISSI-subscale, score 0–16; ^g ^Two items, total score 2–10; ^h ^ISSI-subscale, score 0–30;

Raw scores of assessed HRQOL in the SF 36, demonstrated that the UCP patients, both men and women, perceived their HRQOL lower than their referents (Table 5). Odds Ratio calculated on all the subscales showed that patients with UCP were two to five times more likely to have low scores of HRQOL (Table 6).

**Table 5:** Health-related quality of life (HRQOL) in patients with unexplained chest pain (UCP) and referents.

	**MEN**			**WOMEN**		
		
**Subscales SF 36**^a^	UCP n = 127	Referents n = 490	p-value	UCP n = 104	Referents n = 579	p-value
Physical functioning, mean	84,7	90,9	0.001	73,0	88,1	<.0001
Role physical, mean	75,2	86,6	<.0001	68,8	83,1	<.0001
Vitality, mean	55,2	78,5	<.0001	53,6	71,5	<.0001
Bodily pain, mean	66,9	73,1	<.0001	60,0	72,4	<.0001
General health, mean	51,7	65,8	0.006	44,5	58,8	<.0001
Social functioning, mean	73,4	84,3	<.0001	66,5	79,6	<.0001
Role emotional, mean	78,8	87,1	0.001	72,7	84,9	<.0001
Mental health, mean	68,5	77,9	<.0001	59,9	73,3	<.0001

^a ^Scale score 0–100

**Table 6:** Health-related quality of life (HRQOL) in patients with unexplained chest pain (UCP) and referents.

	**MEN**							**WOMEN**						
		
	UCP n = 127		Referents n = 490					UCP n = 104		Referents n = 579				
						
**Subscales SF 36**	%	(n)	%	(n)	p-value	OR	95% CI	%	(n)	%	(n)	p-value	OR	(95% CI)
Physical functioning, index score ≤ 80^a^	31	(39)	16	(77)	<.0001	2.5	(1.6–3.9)	49	(47)	24	(137)	<.0001	3.1	(2.0–4.8)
Role physical, index score ≤ 69^a^	38	(46)	18	(85)	<.0001	2.9	(1.9–4.5)	51	(49)	23	(133)	<.0001	3.5	(2.2–5.4)
Vitality, index score ≤ 38^a^	32	(39)	14	(67)	<.0001	2.9	(1.8–4.6)	42	(41)	22	(125)	<.0001	2.6	(1.7–4.1)
Bodily pain, index score ≤ 50^a^	41	(50)	12	(58)	<.0001	5.1	(3.3–8.1)	50	(48)	23	(132)	<.0001	3.3	(2.1–5.2)
General health, index score ≤ 55^a^	32	(39)	20	(96)	0.005	1.9	(1.2–2.9)	40	(38)	21	(121)	<.0001	2.5	(1.6–3.9)
Social functioning, index score ≤ 50^a^	28	(35)	14	(67)	0.0001	2.5	(1.6–4.0)	39	(38)	20	(117)	<.0001	2.5	(1.6–3.9)
Role emotional, index score ≤ 67^a^	30	(35)	17	(80)	0.001	2.2	(1.4–3.5)	39	(37)	20	(115)	<.0001	2.6	(1.6–4.1)
Mental health, index score ≤ 60^a^	38	(47)	18	(87)	<.0001	2.8	(1.8–4.4)	53	(52)	24	(140)	<.0001	3.5	(2.3–5.5)

^a ^Lowest quintile in the total referent group, scale score 0–100

## Discussion

In this study, in which UCP patients were compared with a reference group from the general population, we found that the patients differed from the reference group in several respects. After adjusting for differences in age, smoking, hypertension and diabetes, UCP patients were more likely than the referents to be immigrants, perceived more stress at work and had more symptoms of depression and trait-anxiety.

Twice as many UCP patients, compared with reference group, were immigrants, despite the fact that one in ten of the potentially eligible UCP patients was excluded due to language difficulties. One reason for the large number of immigrants admitted to the ED might be that immigrants are not entirely familiar with the Swedish health-care organisation. Instead of seeking care for UCP in primary health care, they sought care at the ED. Also the combination of chest pain, higher levels of risk factors and symptoms of depression and anxiety in the UCP patients in general, might be a reason to contact the ED for investigation.

In agreement with the present study, it has previously been found that immigrants were likely to have more cardiovascular risk factors than Swedish-born individuals, but the incidence of MI was equal in Swedish-born individuals and immigrants. The decreasing trend in coronary heart disease (CHD) in Sweden [17] is unlikely to be explained by the increasing number of immigrants.

The present study revealed differences in educational level between the patients, particularly the women, and the reference group. Twice as many female referents compared with cases had a university education, which is an important finding, as previous studies have indicated that education may be a protective factor for cardiovascular disease [18,19].

The overall prevalence of smoking among men, both cases and referents, as well as in female reference group, was low, reflecting the overall low prevalence of smoking in Sweden, whereas one in three women with UCP was an active smoker. However, after taking differences in education and other factors into account, smoking did not differ between female cases and referents.

Furthermore, UCP patients, both men and women, reported lower alcohol consumption, an unexpected finding, because UCP is associated with anxiety, depression and impaired mental well-being, which, in turn, is a risk for high alcohol consumption [20]. Previous studies investigating the connection between cardiovascular diseases and alcohol consumption in men and women indicated that moderate alcohol consumption might be a protective factor [20-22]. Why this should extend to chest pain patients is unclear.

From 1985 to 2002, the prevalence of overweight and obesity in the general population increased, particularly in men, while leisure activity increased in women [10]. Both inactivity and obesity are well-known risk factors for IHD [23]. Our results suggest that obese and sedentary individuals may also be at risk of UCP. An alternative explanation for the higher levels of traditional risk factors might be that some patients, despite a lack of objective signs still have had undetected IHD. A large percentage of unrecognised myocardial infarction – 19% – was found in a sample of Swedish men and women aged 70 [24].

UCP patients in the present study reported perceived stress at work (constant during the last year or the last five years) to a higher extent than the reference group. This has previously been found in a qualitative study of UCP patients [8]. The results are also in agreement with a study by Jerlock et al. [25] investigating stress at work among UCP patients where 18% of patients reported permanent stress. Future research should address the question of whether patients suffering from UCP have more stressful work environments or a poorer ability to cope with stress in general.

The present study indicates that almost twice as many UCP patients reported higher scores for symptoms of depression and trait-anxiety. The associations between chronic pain and depressive symptoms in an elderly population have previously been studied, with significant associations between pain experience and depressive symptoms, particularly in men. In that study, women consumed more antidepressants and reported a higher level of daily pain [26]. When comparing patients with ischemic heart disease with patients with non-specific chest pain, Tew [27] found that the latter appeared to be more anxious and worried more about their symptoms. Another study evaluating non-cardiac chest pain found that 57% of the included patients suffered from depression and anxiety and suggested that possible psychiatric disorders could be identified since impaired quality of life and pathological coping strategies were found in these patients [28]. Likewise, in the present study, the UCP patients reported a lower HRQOL, in agreement with other earlier studies, indicating that patients with chest pain have impaired quality of life [29-32]. The effect of childhood adversity, mental distress and lack of social support might also have an effect on perceived HRQOL in patients with non-cardiac chest pain, demonstrated in a study of Biggs et al [33].

Esler and Bock [34] claim that a new approach to treating patients suffering from non-cardiac chest pain is needed and recommend a brief intervention delivered at the ED, based on the biopsychosocial model, in order to reduce health-care utilisation, reduce psychological distress and improve health-related quality of life. Atienza et al stress that using a specific questionnaire; a reliable and sensitive tool, when assessing quality of life in chest pain patients, is most desirable [35]. Furthermore, guidelines for the treatment of chest pain patients at the ED could potentially improve the clinical decision-making and reduce the re-admission of patients with cardiac or possible cardiac chest pain [36].

Patients with medically unexplained symptoms include a great number of all patients seeking care. Several of theses patients have culturally founded models explaining their somatic symptoms, and research has demonstrated that e.g. pain experience can be expressed in various ways in different cultures [37,38]. This has to be considered in the care of the immigrants among the UCP patients. In accordance with findings in our study, Kirmayer & Yong (1998) found that patients with e.g. panic disorder or symptoms of depression and anxiety also present somatic symptoms [39].

In present study there were few gender differences between UCP patients and referents: UCP women had less education and more symptoms of depression, UCP men perceived more often stress at work and reported more sedentary in leisure time. These gender differences might be explained by the number of immigrants among UCP patients. According to Bendelow, gender differences in perception of pain can be affected of different factors like sex roles and cultural socialisation [40].

There are several limitations to this study. One inherent feature of a case-reference study is the problem of determining what is cause and what is effect, e.g. are the UCP patients less active because they have chest pain or the reverse? Secondly, the data relating to the UCP patients were relatively small, only collected during office hours Monday through Friday. Only patients who understood Swedish were included, because of the need to understand Swedish when filling in the questionnaire. This may have influenced our results, but we believe it is unlikely that patients with UCP who were immigrants or who sought care during out-of-office hours had lower levels of stress and other psychosocial factors than the UCP patients who were included. Thirdly, the UCP female sample was relatively small, limiting the statistical power of our analyses. The results showed that the male UCP patients were younger than their referents. A further limitation may be that persons <25 years old were not included due to the reference group (25–69 years). In addition, self-reported data for height and weight were used for the UCP patients but not for the reference group. It is therefore likely that the differences in BMI and obesity are even larger, since the under-reporting of weight and height is well documented [41,42]. Finally, a proportion of the referents, or 26%, did not complete all the questionnaires. The non-completers were somewhat older, with less education, which could have exaggerated differences between chest pain cases and the reference group.

## Conclusion

In this study, we found that UCP patients were likely to be immigrants, perceived more stress at work and had more symptoms of depression and trait-anxiety, compared with the participants in the reference group. After adjustment for differences in age, smoking, hypertension and diabetes, these factors were still significantly more common among patients with UCP. The results of the present study indicate that matters relating to work situation, psychosocial factors and to be an immigrant, may be of importance in the patient's decision to seek care for their chest pain. Further research should be directed not only at more efficient ways of identifying organic causes of chest pain but also to a more systematic evaluation of their symptoms, or the potential effects of lifestyle counselling in this large patient population.

## Competing interests

The authors declare that they have no competing interests.

## Authors' contributions

AJF, AR and CW contributed to the design. AJF collected the data in cases. LL was responsible for the collection of psychosocial data in the reference sample. AJF, KIK and CW carried out the data analysis and interpreted the data. AJF wrote the manuscript. KIK, AR, LL, KM and CW revised the manuscript for important intellectual content. All the authors have read and approved the final manuscript.

## Pre-publication history

The pre-publication history for this paper can be accessed here:


